# Two Cases of Interstitial Keratitis with Teeth of the Heredito-Syphilitic Type

**Published:** 1860-07

**Authors:** 


### ARTICLE XXI.

### *Two Cases of Interstitial Keratitis with Teeth of the Heredito-Syphilitic Type.*

Any one who has access to St. Bartholomew's Hospital, and who is desirous to make himself acquainted with the dental malformations indicative of hereditary syphilis, may examine two typical examples of them in patients now in Darker Ward. Both the patients are boys, aged fifteen, and both are under care for interstitial keratitis. According to the views now entertained at the Opthalmic Hospital, this form of inflammation of the cornea occurs almost solely in the subjects of inherited syphilis, and it is a matter of established clinical observation, that those who suffer from it, almost invariably exhibit remarkable peculiarities in their teeth. Occasionally it occurs before the second dentition, and then the teeth are no guide in diagnosis, as the peculiarities are manifested only in the permanent set. The malformations to which we refer mostly effect, in greater or less degree, all the incisor and canine teeth of both jaws, but the central incisors of the upper jaw are those most commonly and most peculiarly modified. These latter may indeed be regarded as the test teeth in reference to this question, and any one not familiar with abnormal dental types will do well to confine his attention to them in attempting to establish a diagnosis as to inherited syphilis.

"Our immediate object in alluding to this subject at the present time is simply to draw attention to Mr. Stanley's two cases, for it is not often that such well-marked ones are met with in the wards of our general hospitals. At the opthalmic hospitals, syphilitic teeth may be seen every day, but it is because their subjects are brought there on account of the keratitis from which they usually suffer at or near the period of puberty.

The following are brief particulars of the cases to which we refer. It will be seen that we have not been able to obtain any history from the patient in either instance. It is, however, one of the great advantages of a knowledge of the typical form of teeth, that it enables its possessor to dispense with the necessity for asking direct questions, which always occasion annoyance, and are usually answered without any regard to the truth.

"CASE 1.—*Double Interstitial Keratitis of much Severity at the age of Thirteen. Characteristic Teeth. History of Eye Disease of an Elder Sister.*—W. L., aged fifteen, is a tall well-grown lad. The bridge of his nose is not much widened or sunken. His forehead is slightly protuberant, and his face shows patches of psoriasis. His palate is very high and narrow. He is rather deaf. He states that he is the second of three children. An elder sister, aged eighteen, has suffered from 'bad eyes,' and is now quite blind of one. A younger sister, aged thirteen, is believed to be in good health.

"*State of Eyes.*—Both corneæ are extensively opaque by the interstitial deposit of lymph. They are also, as is not unusual after a long and severe attack, thinned in the centre, and pushed forward so as to be more convex than is normal. The disease is now slowly subsiding, and the clouds of opacity are gradually being absorbed. The left eye was the first attacked, and its fellow began to inflame about eighteen months ago. The right was not affected until three months later, but they have since suffered with almost equal severity.

"*State of Teeth.*—The central upper incisors are very peculiar indeed, and are of the form most unmistakably typical. Instead of becoming wider from side to side as they project downward, they are narrowed and their corners are rounded off. In the centre of their cutting edge is a deep vertical notch which is prolonged upward as a shallow groove in the middle of the front surface of the tooth. The teeth are carious at their necks. The lateral incisors are so much destroyed by caries that their original form is not recognizable. The lower teeth are all of good form, with the exception of the central incisors, which are narrow, peg-like, and notched.

"CASE 2.—*Double Interstitial Keratitis at the Age of Twelve. Syphilitic Physiognomy. Characteristic Teeth.* —George M., aged fifteen. This lad is of pale complexion, broad, sunken nose, and flabby skin. He is the eldest living of a family of five—two older than himself having died in infancy (one at six months and one at two months.) He has suffered for several years from purulent discharge from both ears, and is now partially deaf.

"*State of Eyes.*—His right eye was first attacked about three years ago, and the left some time subsequently. The left cornea appears never to have suffered so severely as the other, and is now clear, excepting a slight haze in its lower part. He can see to read with the left, but is troubled with muscæ and black spots. The right cornea is still very extensively diseased, the masses of deposit being unusually large and dense.

"*State of Teeth.*—The central upper incisors present each a broad notch in their cutting edges. They are not particularly narrowed, and though not nearly so peculiar in form as those in William L's case (see above,) they still present the same feature (an atrophy of the middle lobe of the tooth-edge,) and are sufficiently characteristic. The lower incisors are serrated, but not much notched.

"Among Mr. Dixon's out patients at the Opthalmic Hospital there are at present many very good examples of the syphilitic type of teeth in conjunction with interstitial keratitis. In one instance three children (a sister and two brothers) are all affected by the same form of disease, and all present the same type of dental abnormities. It is most interesting, however, to observe the gradations in severity according to the age of the patient, both in regard to the eyes and the teeth. The girl is the eldest living of the family, and her teeth have suffered most severely, while her countenance is repulsively deformed by the effects of infantile syphilis, her forehead being protuberant, bridge of nose level with cheeks, large scars at angle of mouth, etc. Her next brother presents characteristic teeth, and has suffered a well-marked attack of keratitis, but his physiognomy is not very peculiar, while the third presents, both as regards eyes and teeth, a still less well-marked condition of things. They have three younger brothers who are reported healthy. The history given is that both father and mother went through the ordinary course of constitutional syphilis and mercurial treatment soon after marriage, and that their two eldest both died in infancy of inherited disease. The girl (their third) is stated to have suffered severely from the usual symptoms in infancy, but got through them. Neither of her brothers are remembered to have had anything noticeable in infancy. It is worthy of remark, in relation to the following case, that the girl is deaf, partially paralytic in her right arm, and almost an idiot as regards intellect."

[*Med. Times and Gaz.*

